# *Pseudomonas*-Derived Ceramidase Induces Production of Inflammatory Mediators from Human Keratinocytes via Sphingosine-1-Phosphate

**DOI:** 10.1371/journal.pone.0089402

**Published:** 2014-02-25

**Authors:** Ami Oizumi, Hitoshi Nakayama, Nozomu Okino, Chihiro Iwahara, Katsunari Kina, Ryo Matsumoto, Hideoki Ogawa, Kenji Takamori, Makoto Ito, Yasushi Suga, Kazuhisa Iwabuchi

**Affiliations:** 1 Institute for Environmental and Gender-specific Medicine, Juntendo University Graduate School of Medicine, Urayasu, Japan; 2 Department of Dermatology, Juntendo University Urayasu Hospital, Urayasu, Japan; 3 Laboratory of Biochemistry, Juntendo University School of Health Care and Nursing, Urayasu, Japan; 4 Department of Bioscience and Biotechnology, Graduate School of Bioresource and Bioenvironmental Sciences, Kyushu University, Fukuoka, Japan; Ben-Gurion University of the Negev, Israel

## Abstract

Ceramide is important for water retention and permeability barrier functions in the stratum corneum, and plays a key role in the pathogenesis of atopic dermatitis (AD). A *Pseudomonas aeruginosa*-derived neutral ceramidase (PaCDase) isolated from a patient with AD was shown to effectively degrade ceramide in the presence of *Staphylococcus aureus*-derived lipids or neutral detergents. However, the effect of ceramide metabolites on the functions of differentiating keratinocytes is poorly understood. We found that the ceramide metabolite sphingosine-1-phosphate (S1P) stimulated the production of inflammatory mediators such as TNF-α and IL-8 from three-dimensionally cultured human primary keratinocytes (termed “3D keratinocytes”), which form a stratum corneum. PaCDase alone did not affect TNF-α gene expression in 3D keratinocytes. In the presence of the detergent Triton X-100, which damages stratum corneum structure, PaCDase, but not heat-inactivated PaCDase or PaCDase-inactive mutant, induced the production of TNF-α, endothelin-1, and IL-8, indicating that this production was dependent on ceramidase activity. Among various ceramide metabolites, sphingosine and S1P enhanced the gene expression of TNF-α, endothelin-1, and IL-8. The PaCDase-enhanced expression of these genes was inhibited by a sphingosine kinase inhibitor and by an S1P receptor antagonist VPC 23019. The TNF-α-binding antibody infliximab suppressed the PaCDase-induced upregulation of IL-8, but not TNF-α, mRNA. PaCDase induced NF-κB p65 phosphorylation. The NF-κB inhibitor curcumin significantly inhibited PaCDase-induced expression of IL-8 and endothelin-1. VPC 23019 and infliximab inhibited PaCDase-induced NF-κB p65 phosphorylation and reduction in the protein level of the NF-κB inhibitor IκBα. Collectively, these findings suggest that (i) 3D keratinocytes produce S1P from sphingosine, which is produced through the hydrolysis of ceramide by PaCDase, (ii) S1P induces the production of TNF-α via S1P receptors, and (iii) released TNF-α stimulates the production of inflammatory mediators such as IL-8.

## Introduction

Atopic dermatitis (AD) is the most common chronically relapsing allergic skin inflammatory disease. The diagnosis of AD is based on its characteristic distribution of eczema, itches, and cutaneous hyperreactivity [1]. Recent findings have shown that disruption of immune epithelial barrier systems are involved in the pathogenesis of immune disorders in AD [2], [3]. The epidermis is composed primarily of proliferating and differentiating keratinocytes arranged in four distinct cell layers: the basal, prickle, and granular cell layers and the stratum corneum [4]. The stratum corneum is composed of terminally differentiated keratinocytes interspersed with intercellular lipids that are involved in both water retention and permeability barrier functions [5]. Ceramides account for 30–40% of stratum corneum lipids, as well as being the major water-retaining molecules and the major binders of structural proteins in the extracellular space of the stratum corneum. [6], [7]. Ceramides are markedly reduced in the stratum corneum of AD skin, irrespective of the presence of lesions [8], and the skin of most patients with AD is colonized by *Staphylococcus aureus* (*S. aureus*) [9]. Decreases in ceramides may lead to increased transepidermal water loss, contributing to the dry and cracked skin that predisposes to *S. aureus* colonization [10]. *Pseudomonas aeruginosa* (*P. aeruginosa*) is another suspected microfloral constituent of atopic skin [11]. A neutral CDase from *P. aeruginosa* AN17 (PaCDase) has been isolated from a patient with AD [12]. *S. aureus* contains cardiolipin and phosphatidylglycerol, which enhance PaCDase hydrolysis in AD skin [13]. Several strains of *P. aeruginosa*, including AN17, secrete significant amounts of staphylolytic proteases that lyse *S. aureus* cells, resulting in their release of cardiolipin and phosphatidylglycerol. Thus, it is likely that PaCDase hydrolyzes ceramide in atopic skin.

The hydrolysis of ceramide by CDase yields sphingosine and fatty acids. Sphingosine can be phosphorylated by sphingosine kinase (SphK) to form sphingosine-1-phosphate (S1P), a molecule involved in a wide range of cellular functions, including growth, differentiation, survival, chemotaxis, angiogenesis, and embryogenesis, in various types of cells [14], [15]. S1P has been shown to be involved in the immunological functions of T lymphocytes and Langerhans cells in AD [15]–[18]. These observations suggest that ceramide metabolites, particularly S1P, are involved in AD. Thus, the topical use of S1P and other sphingosine compounds is under investigation [17]. A recent study demonstrated that SIP was produced by ER stress and mediated the generation of cathelicidin, an antimicrobial peptide, in human keratinocytes [19]. S1P has been shown to inhibit keratinocyte proliferation, promote corneocyte differentiation, and chemoattract keratinocytes [15]. The metabolic conversion of ceramide to S1P protects keratinocytes against UVB-induced, ceramide-mediated apoptosis [20]. However, little is known regarding the role of ceramide metabolites in the global immunological functions of differentiating keratinocytes. A three-dimensional culture system of keratinocytes has been developed that simulates epidermal differentiation at its air-liquid interface, including the generation of basal, spinous, and granular layers and a stratum corneum. The stratum corneum in this system displays permeability barrier functions [21]. This study evaluated the effects of PaCDase on gene expression and the production of inflammatory cytokines and chemokines by three-dimensionally cultured human primary keratinocytes (hereafter termed “3D keratinocytes”).

## Materials and Methods

### Reagents

Sphingosine was purchased from Biomol (Plymouth Meeting, PA, USA). 2-Hydroxy-tetradecanoic acid (α-hydroxy myristic acid) and phytosphingosine were from Matreya (Pleasant Gap, PA, USA). N-acetyl-D-erythro-phytosphingosine, S1P receptor antagonist (VPC 23019), and its negative control (TFA salt) were from Avanti Polar Lipids (Alabaster, AL, USA). The sphingosine kinase inhibitor (SphK inhibitor) [2-(p-hydroxyanilino)-4- (p-chlorophenyl) thiazole, HCl] and S1P were from Calbiochem (Darmstadt, Germany). Phosphatidylglycerol, cardiolipin, curcumin and anti-β-actin antibody were from Sigma-Aldrich (St. Louis, MO, USA). Infliximab (an antibody that binds TNF-α) was from Mitsubishi Tanabe Pharma (Tokyo, Japan). Normal human IgG was from Bethyl Laboratories (Montgomery, TX, USA). Biotin-labeled-RNA sense and anti-sense probes were from Genostaff (Tokyo, Japan). Anti-NF-κB p65 (L8F6), anti-phospho-NF-κB p65 (Ser536), anti-TNF-α and anti-IκBα antibodies were from Cell Signaling Technology (Danvers, MA, USA). Anti-human SphK1 antibody was from R & D Systems (Minneapolis, MN, USA).

Recombinant *Pseudomonas*-derived ceramidase (PaCDase; 16700 mU/ mg protein) [12] and its mutant H97A/H99A-PaCDase [22], which has enzyme activity 576-fold lower than that of PaCDase (29 mU/mg protein), were produced as described [12]. The PaCDase concentration used throughout the study was 50 pg/ml (1 mU/ml).

### Three-dimensional cell culture and stimulation of human primary keratinocytes

The LabCyte EPI-Model (J-Tec Co., Gamagori, Japan), consisting of normal human epidermal keratinocytes cultured to form a multilayer, provides a highly differentiated model of the human epidermis [23], . The stratum corneum in this system displays permeability barrier functions.

To evaluate the effects of CDase and other reagents on keratinocyte functions, we applied an epicutaneous 24-h patch test on the EPI-Model in keratinocyte serum-free medium (keratinocyte-SFM) (Invitrogen; Carlsbad, CA, USA), as described [25], with several modifications. Briefly, sterilized nitrocellulose filters (diameter 10 mm, pore size 3.0 µm; Sartorius Stedim Biotech GmbH; Goettingen, Germany) were incubated with PaCDase or other reagents in 25 mM Tris-buffered saline (pH 8.5), 2.5 mM CaCl_2_, with or without 0.1% (1.6 mM) Triton X-100, for 4 h at 4°C. Excess liquid was removed by blotting with sterile filter paper, and the wetted filters were placed onto the stratum corneum of the EPI-Model in 10-mm diameter chambers and incubated in a CO_2_ incubator for 24 h. Various inhibitors and antibodies were added to both the filter soaking solution and the culture medium to determine their effects on PaCDase-induced keratinocyte responses. In some experiments, the cells were incubated for 30 min or 4 h to determine the expression of proteins.

#### Total RNA preparation

The membranes of cultured cells were removed from the chambers and treated with TRIzol reagent (Invitrogen). Total RNA was isolated using an Absolutely RNA Purification Kit (Agilent Technologies; Palo Alto, CA, USA) according to the manufacturer's protocol.

### Quantitative real-time RT-PCR

Complementary DNA was synthesized from 50 ng/l total RNA using an ExScript RT-PCR Kit (Takara-Bio, Shiga, Japan). Primers were selected using the Perfect Real-Time Primer Support System (Takara-Bio). Real-time RT-PCR was performed using SYBR Premix Ex Taq (Takara-Bio) and an ABI 7900HT Sequence Detector System (Applied Biosystems; Foster City, CA). The amplification program consisted of initial denaturation at 95°C for 10 s, followed by 40 cycles of denaturation at 95°C for 10 s, and annealing and extension at 60°C for 30 s. Dissociation curves were plotted to determine the specificity of the PCR products. Relative cDNA concentrations were determined using standard curves generated from sequential 10-fold dilutions of cDNA synthesized from QPCR Human Reference Total RNA (Stratagene; La Jolla, CA). All results were normalized relative to 18S ribosomal protein as an internal control.

### Microarray analysis

cDNA was synthesized and aminoallyl-labeled RNA (aaRNA) produced using the Amino Allyl Message Amp II™ aaRNA Amplification Kit (Applied Biosystems), according to the manufacturer's protocol. Cy3- and Cy5-labeled aaRNA were concentrated using Microcon YM-30 centrifugal filter units (Nihon Millipore K.K., Tokyo, Japan), mixed with the hybridization buffer supplied with the kit, and denatured at 95°C for 2 min. The hybridization mixture was applied to a “3D-Gene” human oligo chip 25k (Toray Industries Inc., Tokyo, Japan) and incubated according to the manufacturer's instructions. The DNA chip slides were washed and dried, and the fluorescent signals were quantified using a GenePix 4400a (Molecular Devices; Sunnyvale, CA, USA) and analyzed using GenePix Pro7 software (Molecular Devices). Background values were subtracted from the raw intensity values, and the resulting values were normalized by the global normalization method [26].

Genes with a fluorescence intensity ≥10-fold higher than background, based on the results of dye-swapping experiments, were considered positively expressed. The microarray data are available at the National Center for Biotechnology Information's Gene Expression Omnibus site (which can be accessed at www.ncbi.nlm.nih.gov/geo) accession no. GSE53670.

### *In situ* hybridization for TNF-α

Membranes cut from chambers of the EPI-Model and containing cultured cells were embedded in paraffin and sectioned at a thickness of 4 µm. The sections were de-waxed with xylene, rehydrated through an ethanol series and PBS, fixed with 4% paraformaldehyde, incubated with a peroxidase-blocking reagent (0.3% hydrogen peroxide; Dako Corp.; Carpinteria, CA, USA) for 15 min, rinsed with PBS, treated with 10 µg/ml proteinase K, washed with PBS, placed in 0.2 N HCl for 10 min, and washed again. The sections were then hybridized at 55°C for 16 h with 300 ng/ml biotin-labeled probes in probe diluent (Genostaff), washed in HybriWash (Genostaff), treated with RNase, treated for 30 min with streptavidin-HRP from an LSAB+ Kit (Dako), washed with PBS, incubated with 3,3′-diaminobenzidine (DAB), counterstained with hematoxylin, and covered with cover slips.

### Measurement of ceramide, sphingosine and S1P

Amounts of sphingosine and S1P were measured by HPLC (HITACHI L-7110 HPLC system, Hitachi High-Technologies) after derivatization with *o*-phthalaldehyde (OPA) as described [27]. To measure of ceramide, samples were incubated with PaCDase, and the produced sphingosine contents were determined as described [28], with modifications.

### Immunohistochemistry

Membrane sections as described above were incubated with 0.3% hydrogen peroxide for 15 min, rinsed with PBS, blocked with 10% normal swine serum in PBS for 20 min, and incubated with anti-human TNF-α polyclonal rabbit IgG (Santa Cruz Biotechnology; Santa Cruz, CA, USA), anti-endothelin-1 rabbit IgG (Medical Biological Laboratories, Gunma, Japan), and/or anti-human IL-8 monoclonal mouse IgG (Proteintech Group Inc.; Chicago, IL, USA). The sections were subsequently washed with PBS, incubated with secondary antibody (EnVision+ System-HRP-Labeled Polymer; Dako) for 1 h, washed with PBS, incubated with DAB, counterstained with hematoxylin, and observed with a light microscope (model BX51; Olympus; Tokyo, Japan).

### Western blotting

Levels of phosphorylated NF-κB p65, IκBα, and β-actin were analyzed by western blotting [29]-[31]. The 3D keratinocytes were lysed in lysis buffer (10 mM Tris-HCl, pH 7.5, 50 mM NaCl, 10 mM NaF, 2 mM Na_3_VO_4_, 1 mM DFP, 1% Triton X-100, with 1/20 v/v Complete) and sonicated for 10 s with 30% output using an ultrasonic sonifier (model 250; Branson Ultrasonics; Danbury, CT, USA). The lysates were cleared by centrifugation, and the supernatants were electrophoresed on 10% SDS-PAGE gels under reducing conditions and blotted onto PVDF membranes (Millipore; Billerica, MA, USA). The membranes were incubated with antibodies to phospho-NF-κB p65 (Ser536), IκBα, TNF-α and SphK1. To determine the amount of the antigen proteins in each band, the membranes were reprobed with antibodies to NF-κB p65 or β-actin. The bands were scanned, and the relative signal intensities were quantified using the ImageJ program (National Institutes of Health; Bethesda, MD, USA).

### Statistical analysis

Data were expressed as means±SD and compared by one-way ANOVA using the GraphPad Prism program, V. 5.00 (GraphPad Software Inc.; La Jolla, CA, USA). Differences with *P*<0.05 were considered statistically significant.

## Results

### PaCDase induces TNF-α mRNA in 3D keratinocytes

**Figure 1:**
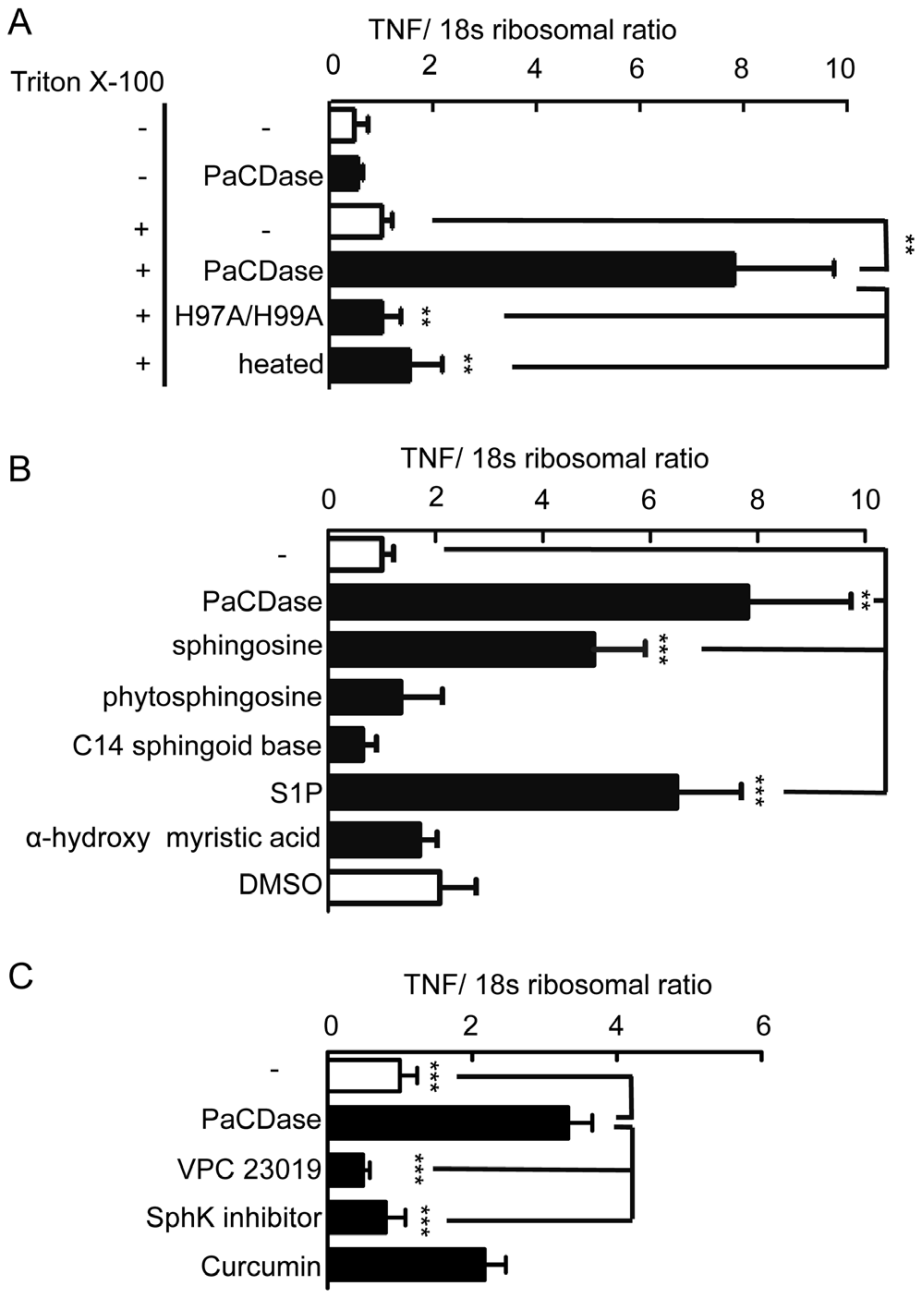
PaCDase enhances TNF-α gene expression via S1P and S1P receptors in 3D keratinocytes. (*A*) PaCDase induces TNF-α gene expression in 3D keratinocytes. Nitrocellulose filters with 1 mU/ml (60 ng/ml) PaCDase, 60 ng/ml H97A/H99A-PaCDase (H97A/H99A), or 60 ng/ml heat-inactivated PaCDase (heated), with or without 0.1% Triton X-100, were placed onto the stratum corneum. The cells were incubated for 24 h, and TNF-α mRNA was assayed by quantitative real-time RT-PCR. Each bar represents the mean±SD of 10 independent experiments. **P<0.01. (*B*) Sphingosine and S1P enhance TNF-α gene expression. Nitrocellulose filters with 1 mU/ml PaCDase, 5 µM sphingosine, phytosphingosine, C14 sphingoid base, S1P, α-hydroxy myristic acid, or 0.1% DMSO (solvent control) in Tris-buffered saline containing 0.1% Triton X-100 were placed onto the stratum corneum. The cells were incubated for 24 h, and TNF-α mRNA was assayed by quantitative real-time RT-PCR and normalized relative to a RNA encoding an S18 ribosomal protein gene. Each bar represents the mean±SD of 5 independent experiments. **P<0.01; ***P<0.001. (*C*) Involvement of SphK and S1P receptor. Nitrocellulose filters with 1 mU/ml PaCDase in the absence or presence of 1 µM VPC 23019, 10 µM SphK inhibitor, or 40 µM curcumin in Tris-buffered saline containing 0.1% Triton X-100 were placed onto the stratum corneum. The cells were incubated for 24 h, and TNF-α mRNA was assayed by quantitative real-time RT-PCR. Each bar represents the mean±SD of 5 independent experiments. *P<0.05; **P<0.01; ***P<0.001.

TNF-α can be released by keratinocytes [32] and is involved in the progression of AD [33]. We therefore evaluated the possible induction of TNF-α mRNA by PaCDase in 3D keratinocytes. Under our experimental conditions, none of the water-soluble stimulants of keratinocytes tested [34], including trypsin, Der P1, and Der f1, affected the expression of any of the genes in our DNA microarray analysis (data not shown). These findings suggest that the stratum corneum of the cell culture model has competent permeability barrier functions against these stimulants. When a nitrocellulose filter containing 2 mU/ml PaCDase was placed onto the stratum corneum of 3D keratinocytes and incubated for 24 h, TNF-α mRNA expression by the keratinocytes was unaltered (Fig. 1A), although the concentration of PaCDase in the assay system was sufficient to degrade ceramide [35]. The non-ionic detergent Triton X-100, which reduces permeability barrier functions, thereby moderately increases transepidermal water loss and the production of erythema on human skin [36]. We therefore used Triton X-100 treated 3D-keratinocytes as a damaged skin model. We found that TNF-α mRNA expression in 3D keratinocytes was increased markedly by PaCDase in the presence of 0.1% Triton X-100 but minimally affected by Triton X-100 alone. TNF-α mRNA expression was not enhanced by heat-inactivated PaCDase or mutant PaCDase. These findings suggest that PaCDase degraded ceramide in the Triton X-100-damaged stratum corneum and that the resulting degradation products enhanced TNF-α mRNA expression by the 3D keratinocytes.

### S1P is involved in TNF-α production by 3D keratinocytes

To identify the types of ceramide metabolites that are responsible for TNF-α production, we assayed the effects of sphingosine, S1P, phytosphingosine, C14 sphingoid base, and α-hydroxymyristic acid on TNF-α mRNA levels in 3D keratinocytes. Of these molecular species, only sphingosine and S1P enhanced TNF-α mRNA levels (Fig. 1B). PaCDase treatment for 1 h slightly, but not significantly, decreased ceramide concentration, and increased sphingosine and S1P concentrations in 3D keratinocyte layers (Table 1). However, the PaCDase-induced expression of TNF-α mRNA in 3D keratinocytes was suppressed not only by a specific SphK inhibitor that inhibits the production of S1P from sphingosine but also by VPC 23019, an antagonist of S1P receptors 1 and 3 (Fig. 1C). The SphK inhibitor alone and VPC 23019 alone minimally affected the TNF-α mRNA levels of the Triton X-100-treated 3D keratinocytes in the absence of PaCDase (data not shown). Therefore, it is likely that S1P is responsible for the PaCDase induction of TNF-α mRNA. The transcriptional regulation of genes encoding inflammatory cytokines, including TNF-α, was shown to be dependent on NF-κB. We found that the NF-κB inhibitor curcumin [37] reduced PaCDase-induced TNF-α expression by 3D keratinocytes by 34.7±17.7% (mean±S.D. of 3 experiments; not significant).

**Table 1:** Effect of PaCDase on ceramide, sphingosine and S1P concentration in 3D keratinocyte layers.

Incubation time	0 h	1 h	24 h
Ceramide			
-	71168±5043	74295±8116	94982±9570
PaCDase	-	70245±3626	91228±6591
Sphingosine			
-	10065±1407	9210±1100	8160±1110
PaCDase	-	9720±920	8150±510
S1P			
-	13.3±7.6	10.6±2.0	7.9±1.8
PaCDase	-	11.7±1.2	7.2±1.3

Nitrocellulose filters with 1 mU/ml PaCDase or 0.1% DMSO (-) in Tris-buffered saline containing 0.1% Triton X-100 were placed onto the stratum corneum. The cells were incubated for 1 h or 24 h. The keratinocyte layers were isolated, and sphingosine and S1P concentrations were measured. The concentrations of ceramide, sphingosine and S1P in the untreated keratinocyte layers are shown as 0 h. Data represent the mean±SD of 3 independent experiments.

**Figure 2:**
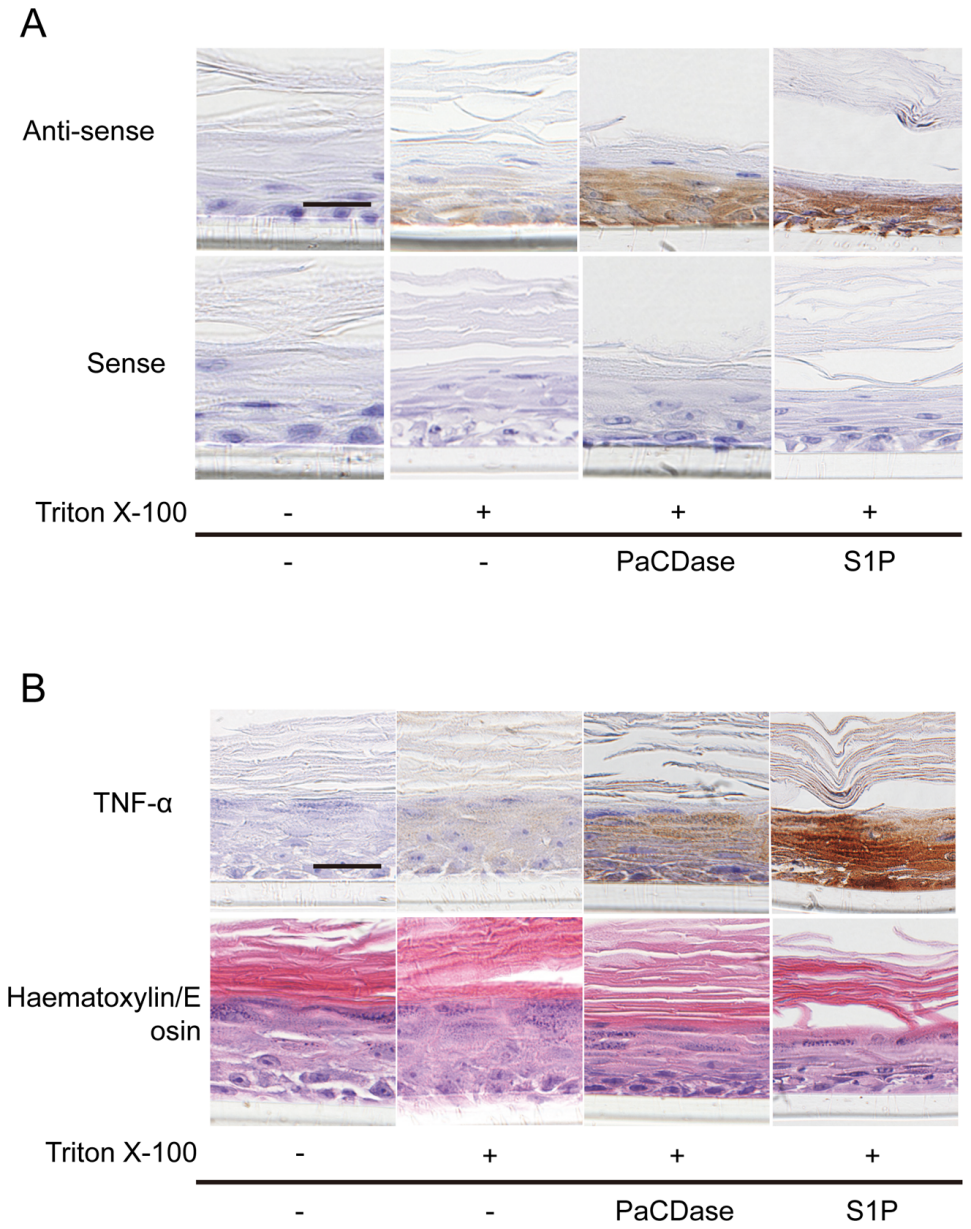
TNF-α is expressed in all layers of PaCDase-treated keratinocytes. (*A*) *In situ* hybridization. Nitrocellulose filters with Tris-buffered saline alone (-/-), without or with 1 mU/ml PaCDase or 5 µM S1P in Tris-buffered saline containing 0.1% Triton X-100 were placed onto the stratum corneum. The cells were incubated for 24 h, embedded in paraffin, and sectioned by cryostat. The sections were fixed with paraformaldehyde and incubated with biotin-labeled-RNA anti-sense and sense probes for TNF-α (x400). The data shown represent 3 independent experiments. Bar: 25 µm. (*B*) Immunohistochemical analysis. Paraformaldehyde-preserved and paraffinized 3D keratinocyte culture sections as described in panel A were incubated with rabbit anti-human TNF-α (upper panel) or with hematoxylin/ eosin (lower panel). The data shown represent 3 independent experiments. Bar: 25 µm.

**Figure 3:**
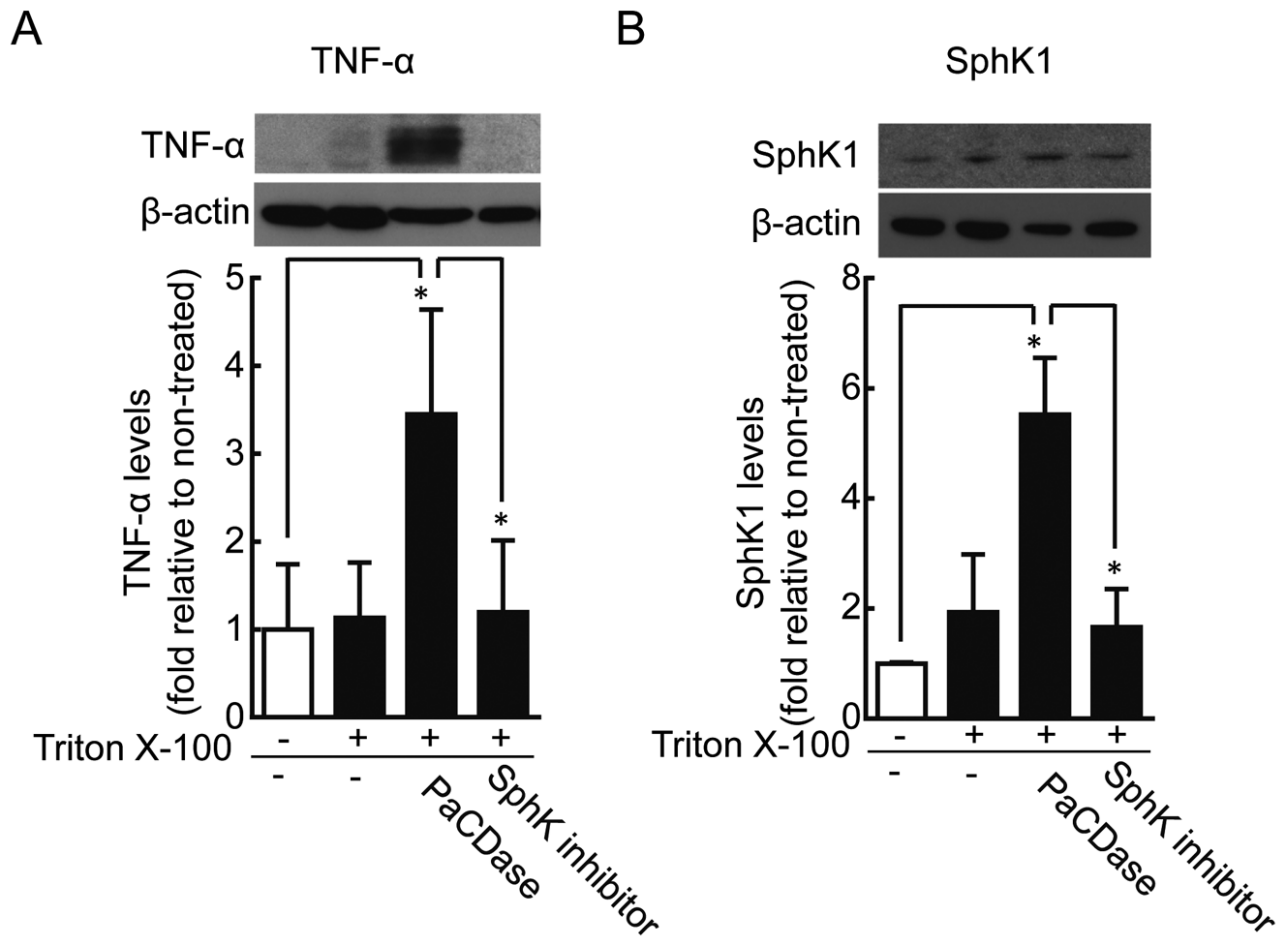
PaCDase-induced production of TNF-α and SphK1 by keratinocytes. PaCDase-induced TNF-α production. Nitrocellulose filters with 1 mU/ml (60 ng/ml) PaCDase without or with 10 µM SphK inhibitor in TBS containing 0.1% Triton X-100 were placed on the stratum corneum. After incubation for 24 h, the cells were washed and solubilized and lysates cleared by centrifugation. The equivalent amounts of protein of the lysates were loaded onto polyacrylamide gels. Membranes were incubated with anti-TNF-α (A) or anti-SphK1 (B) antibody. To determine the amount of membrane-bound form TNF-α or SphK1 in each band, the membranes were re-probed with anti-β-actin. The blots shown are representative of 3 independent experiments. The data are expressed as the ratio relative to control (-/-) and represent the mean±SD of 3 independent experiments. *P<0.05.

To identify the cell layers that produce TNF-α in response to PaCDase, we performed *in situ* hybridization analysis using an antisense TNF-α RNA probe. Positive signals were detected in all layers of PaCDase- and S1P-treated 3D cell cultures but only in the basal layer of Triton X-100-treated cultures (Fig. 2A). Only marginal signals were detected following incubation with a sense RNA probe. Immunohistochemical staining of sections with anti-TNF-α antibody showed that PaCDase and S1P induced TNF-α in all keratinocyte layers of the 3D culture, whereas Triton X-100 alone had only a slight effect (Fig. 2B). Furthermore, western blotting analysis confirmed that PaCDase induced production of TNF-α protein by 3D keratinocytes, and that this production was inhibited by the SphK inhibitor (Fig. 3A).

### PaCDase and S1P induce expression of inflammatory mediators

To investigate the changes in gene expression that occur during treatment of keratinocytes with PaCDase, we performed cDNA microarray analysis. Of the 25,392 probe sets on the “3D-Gene” human oligo chip 25k, 1,125 were regarded as control or background probes. Other genes were eliminated from the analysis if their normalized intensity was less than 2. “Unaltered” genes, *i.e.*, those that showed a <2-fold change in expression level during PaCDase treatment, were also eliminated. This procedure resulted in the identification of 420 genes that were upregulated and 526 that were downregulated in PaCDase-treated 3D keratinocytes. The 20 genes that were most strongly upregulated by PaCDase are listed in Table 2; these include genes that encode endothelin-1, TNF-α, IL-8, claudin-4, CXCL1, GADD45 gamma, TNF-inducible protein, and CTGF. PaCDase treatment also caused a 3.85-fold increase in the level of CXCL2 mRNA. IL-8, CXCL1, and CXCL2 have been reported upregulated in the lesional skin of patients with AD or psoriasis [38]. Claudin-4 expression, which is higher in psoriatic than in normal epidermis [39], was enhanced by PaCDase treatment. In contrast, β-defensins, cathelicidin, IL4, IL5, IL13, RANTES, MCP-4, eotaxin, or CCL-27, all of which were reported to be up-regulated in AD skin [40], were not up-regulated by PaCDase treatment.

**Table 2:** The 20 genes most strongly upregulated by PaCDase in 3D cultured keratinocytes.

Name	ratio	RefSeq_ID	description
NP_853640.1	11.1	NM_181609	Keratin-associated protein 19-3
KRTAP3-2	11.1	NM_031959	Keratin-associated protein 3-2
DMBX1	8.4	NM_172225	diencephalon/meSensephalon homeobox 1 isoform
KRTAP19-1	8.4	NM_181607	Keratin-associated protein 19-1
EDN1	7.8	-	Endothelin-1
TNF-α	7.6	NM_000594	Tumor necrosis factor
NP_853642.1	7.4	NM_181611	Keratin-associated protein 19-5
IL8	7.0	NM_000584	Interleukin-8
NP_004684.1	6.0	NM_004693	cytokeratin type II
SPOCD1	5.8	-	SPOC domain containing 1
FOS	5.7	NM_005252	Proto-oncogene protein c-fos (Cellular oncogene fos)
KCTD12	5.6	NM_138444	Potassium channel tetramerization domain containing protein 12 (Pfetin)
RHOI_HUMAN	5.6	NM_004675	Rho-related GTP-binding protein RhoI.
CLDN4	5.5	NM_001305	Claudin-4 (Clostridium perfringens enterotoxin receptor)
CXCL1	5.2	-	Growth regulated protein alpha precursor (CXCL1)
JUN	4.9	NM_002228	Transcription factor AP-1 (Activator protein 1)
GADD45G	4.9	NM_006705	Growth arrest and DNA-damage-inducible protein GADD45 gamma
ADAMTS1	4.9	NM_006988	ADAMTS-1 precursor (A disintegrin and metalloproteinase with thrombospondin motifs 1)
TNFAIP6	4.6	NM_007115	Tumor necrosis factor-inducible protein TSG-6 precursor
CTGF	4.5	NM_001901	Connective tissue growth factor precursor (Hypertrophic chondrocyte- specific protein 24).

The results shown are representative data of 3 experiments.

We also identified 513 genes that were upregulated and 756 that were downregulated by S1P. The 20 genes that were most strongly upregulated by S1P are shown in Table 3. The genes that were upregulated by both PaCDase and S1P include those encoding TNF-α, endothelin-1, CXCL1, IL-8, and GADD45 gamma.

**Table 3:** The 20 genes most strongly upregulated by S1P in 3D cultured keratinocytes.

Name	ratio	RefSeq_ID	description
TNF	15.5	NM_000594	Tumor necrosis factor precursor (TNF-alpha)
CXCL1	13.9	-	Growth regulated protein alpha precursor (CXCL1)
EDN1	12.0	-	Endothelin-1 precursor (Preproendothelin-1)
KRTAP3-2	11.3	NM_031959	Keratin-associated protein 3-2
Q9NPD2_HUMAN	10.9	-	Keratin 19
KRT19	10.6	NM_002276	Keratin, type I cytoskeletal 19 (Cytokeratin 19)
DMBX1	10.6	NM_172225	diencephalon/meSensephalon homeobox 1 isoform a
DB105_HUMAN	10.3	NM_152250	Beta-defensin 105 precursor (Beta-defensin 5)
FOS	10.2	NM_005252	Proto-oncogene protein c-fos(Cellular oncogene fos)
HIST1H1BB	9.2	NM_021052	Histone H1B.m (H1B/m)
NP_853640.1	9.2	NM_181609	Keratin-associated protein 19-3
KRTAP19-1	8.6	NM_181607	Keratin-associated protein 19-1
CXCL2	8.4	NM_002089	Macrophage inflammatory protein-1Blpha precursor (CXCL2)
NP_004684.1	8.4	NM_004693	cytokeratin type II
GADD45G	7.9	NM_006705	Growth arrest and DNA-damage-inducible protein GADD45 gamma
IL8	7.7	NM_000584	Interleukin-8 precursor (IL-8)
TFPI2	7.7	NM_006528	Tissue factor pathway inhibitor 2 precursor (TFPI-2)
IRF1	7.7	-	Interferon regulatory factor 1 (IRF-1).
IL23A	7.5	NM_016584	interleukin 23, alpha subunit p19 precursor
NP_853642.1	7.1	NP_853642.1	Keratin-associated protein 19-5

The results shown are representative data of 3 experiments.

cDNA microarray analysis showed that, among the SphKs and S1P receptors tested, PaCDase and S1P selectively enhanced the expression of genes encoding SphK-1 (SPHK1) and S1P3 receptor (Tables 4 and 5). Western blotting confirmed that PaCDase significantly induced production of SphK1 protein by 3D keratinocytes, and that this production was inhibited by SphK inhibitor (Fig. 3B).

**Table 4:** Effect of PaCDase on levels of expression of sphingosine kinase and S1P receptors genes.

Name	-	PaCDase	ratio
SPHK1	2.39	6.40	2.68
SPHK2	0.98	1.07	1.09
S1P(1)/EDG1	0.28	0.28	0.97
S1P(2)/EDG5	0.57	0.42	0.73
S1P(3)/EDG3	0.38	1.00	2.70
S1P(4)/EDG6	0.30	0.40	1.22
S1P(5)/EDG8	0.43	0.23	0.55

Nitrocellulose filters with 1 mU/ml PaCDase or 0.1% DMSO (-) in Tris-buffered saline containing 0.1% Triton X-100 were placed onto the stratum corneum. The cells were incubated for 24 h, and DNA microarray analysis was performed. The results shown are representative of 3 experiments.

**Table 5:** Effect of S1P on levels of expression of genes encoding sphingosine kinases and S1P receptors.

Name	-	S1P	ratio
SPHK1	2.53	5.39	2.13
SPHK2	0.89	1.03	1.16
S1P(1)/EDG1	0.23	0.27	1.18
S1P(2)/EDG5	0.74	0.53	0.71
S1P(3)/EDG3	0.22	0.90	3.99
S1P(4)/EDG6	0.33	0.28	0.85
S1P(5)/EDG8	0.33	0.21	0.64

Nitrocellulose filters with 5 µM S1P or 0.1% DMSO (-) in Tris-buffered saline containing 0.1% Triton X-100 were placed onto the stratum corneum. The cells were incubated for 24 h, and DNA microarray analysis was performed. The results shown are representative data of 3 experiments.

### PaCDase induces endothelin-1 and IL-8 production through S1P

**Figure 4:**
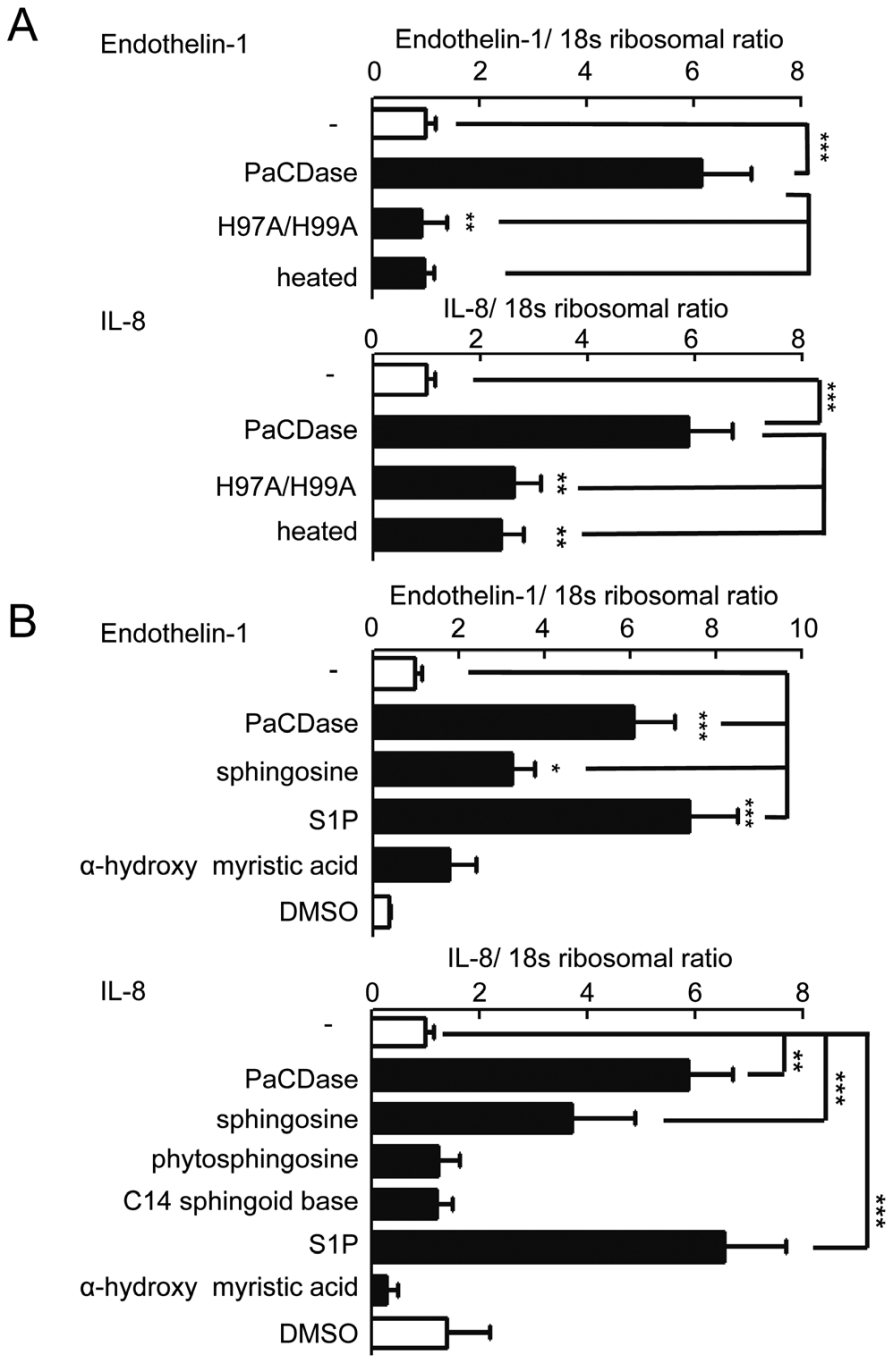
PaCDase-induced endothelin-1 and IL-8 production by keratinocytes. (*A*) Expression of endothelin-1 and IL-8 mRNA is dependent on PaCDase enzymatic activity. Nitrocellulose filters with 1 mU/ml (60 ng/ml) PaCDase, 60 ng/ml mutant H97A/H99A-PaCDase (H97A/H99A), or 60 ng/ml heat-inactivated PaCDase (heated) in TBS containing 0.1% Triton X-100 were placed on the stratum corneum. The cells were incubated for 24 h, and endothelin-1 and IL-8 mRNAs were assayed by quantitative real-time RT-PCR. An S18 ribosomal protein gene was used for normalization. Each bar represents the mean±SD of 5 independent experiments. **P<0.01; ***P<0.001. (*B*) Sphingosine and S1P enhance expression of endothelin-1 and IL-8 mRNA. Nitrocellulose filters without (-) or with 5 µM sphingosine, S1P, or α-hydroxy myristic acid in Tris-buffered saline containing 0.1% Triton X-100 were placed on the stratum corneum. Endothelin-1 and IL-8 mRNAs were assayed by quantitative real-time RT-PCR. Each bar represents the mean±SD of 3 independent experiments. *P<0.05; ***P<0.001.

**Figure 5:**
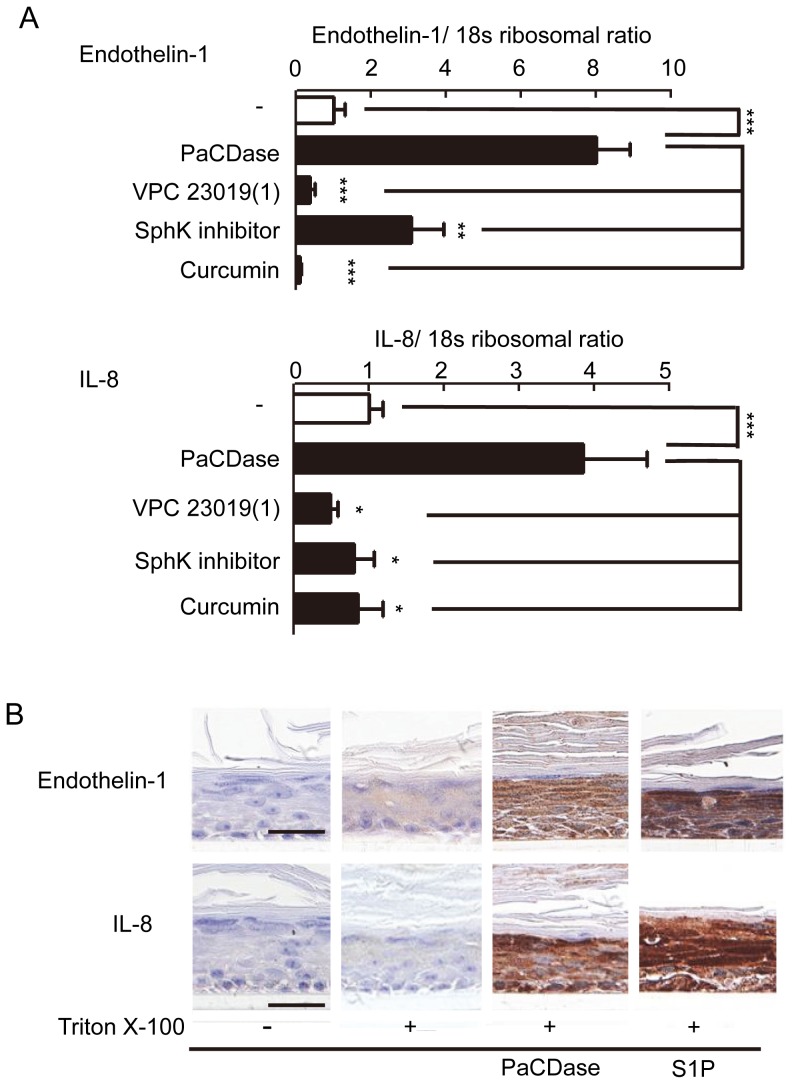
PaCDase-produced S1P induces endothelin-1 and IL-8 production by keratinocytes. (*A*) Involvement of SphK and S1P receptor in PaCDase-enhanced endothelin-1 and IL-8 gene expression. Nitrocellulose filters without (-) or with 1 mU/ml PaCDase in the absence or presence of 1 µM VPC 23019, 10 µM SphK inhibitor, or 40 µM curcumin in Tris-buffered saline containing 0.1% Triton X-100 were placed on the stratum corneum, and endothelin-1 and IL-8 mRNAs were assayed by quantitative real-time RT-PCR. Each bar represents the mean±SD of 3 independent experiments. **P<0.01; ***P<0.001. (*B*) Immunohistochemical analysis. Nitrocellulose filters with Tris-buffered saline alone (-/-), or without (+/−) or with 1 mU/ml PaCDase (+/PaCD) or 5 µM S1P (+/S1P) in Tris-buffered saline containing 0.1% Triton X-100 were placed on the stratum corneum. The cells were incubated for 24 h, embedded in paraffin, sectioned, and incubated with rabbit anti-endothelin-1 IgG (endothelin-s) or mouse anti-human IL-8 IgG. The data shown represent 3 independent experiments. Bar: 25 µm.

**Figure 6:**
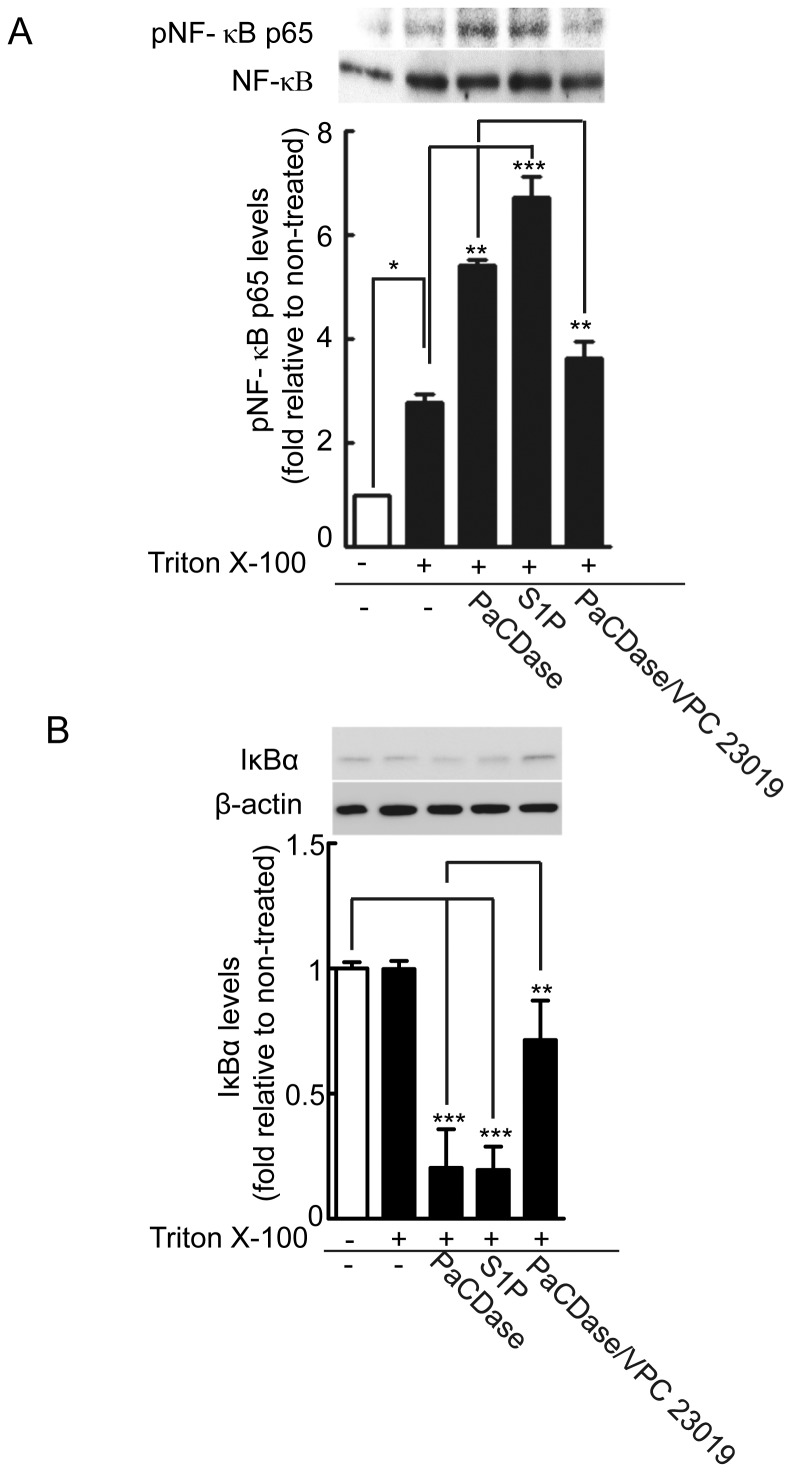
PaCDase and S1P activate NF-κB-dependent signal pathway. (*A*) PaCDase and S1P significantly increase phosphorylated NF-κB p65 levels. Nitrocellulose filters with Tris-buffered saline (-/-) or Tris-buffered saline plus 0.1% Triton X-100 alone (−/+) or with 1 mU/ml PaCDase (PaCD/+), 5 µM S1P (S1P/+), or 1 mU/ml PaCDase with 10 µM VPC 23019 (PaCD/VCP23019/+) were placed onto the stratum corneum and incubated for 4 h. The cells were washed and solubilized, lysates were cleared by centrifugation, and the equivalent amounts of protein of the supernatants were subjected to SDS-PAGE/immunoblotting with anti-phospho-NF-κB p65 (Ser536). To determine the amount of NF-κB p65 in each band, the membranes were re-probed with anti-NF-κB p65. The blots shown are representative of 3 independent experiments. The band intensity of phosphorylated NF-κB p65 is shown relative to that of NF-κB p65 in each lane. The data are expressed as the ratio relative to control (-/-) and represent the mean±SD of 3 independent experiments. *P<0.05; **P<0.01; ***P<0.001. (*B*) PaCDase and S1P decrease IκBα protein concentration in cytosolic extracts in keratinocytes. The cells were incubated for 4 h as described in (A), washed and solubilized, the lysates were cleared by centrifugation, and the supernatants were subjected to SDS-PAGE/immunoblotting with anti- IκBα. To determine the amount of IκBα in each band, the membranes were re-probed with anti-β-actin. The blots shown are representative of 3 independent experiments. The band intensity of IκBα is shown relative to that of βactin in each lane. The data are expressed as the ratio relative to control (-/-) and represent the mean±SD of 3 independent experiments. **P<0.01; ***P<0.001.

Endothelin-1, TNF-α, and IL-8 were the cytokines most strongly upregulated by PaCDase in our 3D keratinocyte culture system (Table 2). The levels of endothelin-1 and IL-8 mRNAs were enhanced by PaCDase, but not affected by mutant or heat-inactivated PaCDase (Fig. 4A). This PaCDase-induced expression of endothelin-1 and IL-8 mRNAs was enhanced by the ceramide-derived lipids sphingosine and S1P (Fig. 4B) and inhibited by VPC 23019 and a SphK inhibitor (Fig. 5A). Immunohistochemical staining of 3D cell culture sections confirmed that PaCDase and S1P induced the production of endothelin-1 and IL-8 proteins from whole 3D keratinocytes (Fig. 5B). Curcumin, which suppresses the TNF-α-induced activation of NF-κB in the human keratinocyte cell line HaCaT [41], inhibited the PaCDase-induced expression of endothelin-1 and IL-8 mRNAs in our system. NF-κB activation is mediated through the activation of specific IκB kinases and the subsequent phosphorylation of IκB. The pathway leading to proteolysis of IκB is denoted as the canonical NF-κB activation pathway [42], [43]. Both PaCDase and S1P significantly enhanced the phosphorylation of NF-κB p65 in 3D keratinocytes, and PaCDase-induced phosphorylation was suppressed by VPC 23019 (Fig. 6A). Concomitant with NF-κB p65 phosphorylation, the level of IκBα protein was markedly reduced by PaCDase and S1P, with the PaCDase-induced reduction suppressed by VPC 23019 (Fig. 6B).

### PaCDase-induced upregulation of IL-8 gene expression is mediated by TNF-α

**Figure 7:**
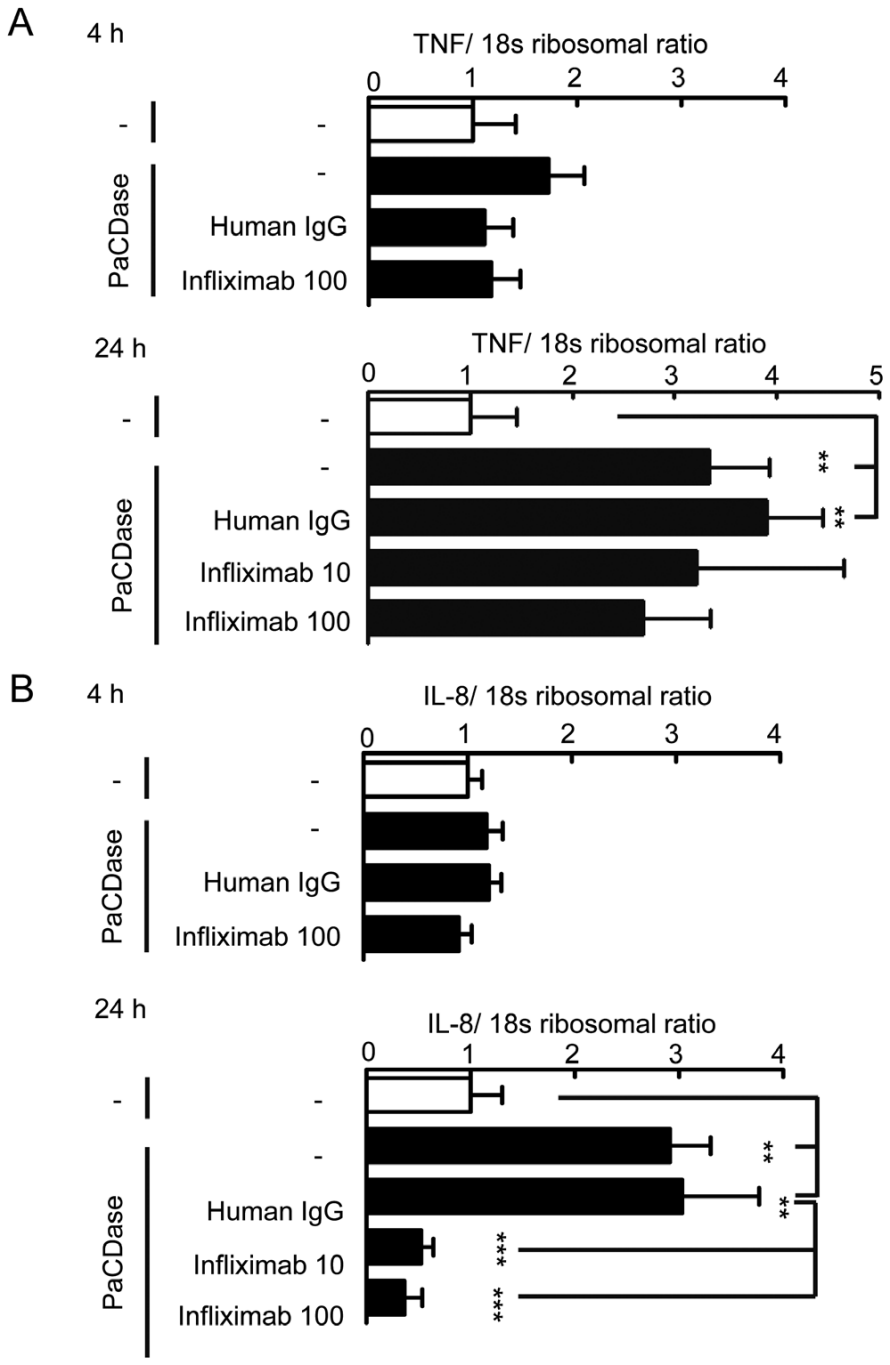
Infliximab inhibits PaCDase-induced production of IL-8, but not TNF-α, by keratinocytes. (*A*) PaCDase-induced TNF-α gene expression is not inhibited by infliximab. Nitrocellulose filters with Tris-buffered saline with 0.1% Triton X-100 (-/-) or with 1 mU/ml PaCDase (-), PaCDase plus 100 µg/ml normal human IgG or 10 or 100 µg/ml infliximab were placed on the stratum corneum. The cells were incubated for 4 h or 24 h, and the level of TNF-α mRNA was determined. An S18 ribosomal protein gene was used for normalization. Each bar represents the mean±SD of 3 independent experiments. **P<0.01. (*B*) Infliximab inhibition of PaCDase-induced IL-8 gene expression. IL-8 mRNA levels were assayed in the samples described in A. Each bar represents the mean±SD of 3 independent experiments. **P<0.01; ***P<0.001.

**Figure 8:**
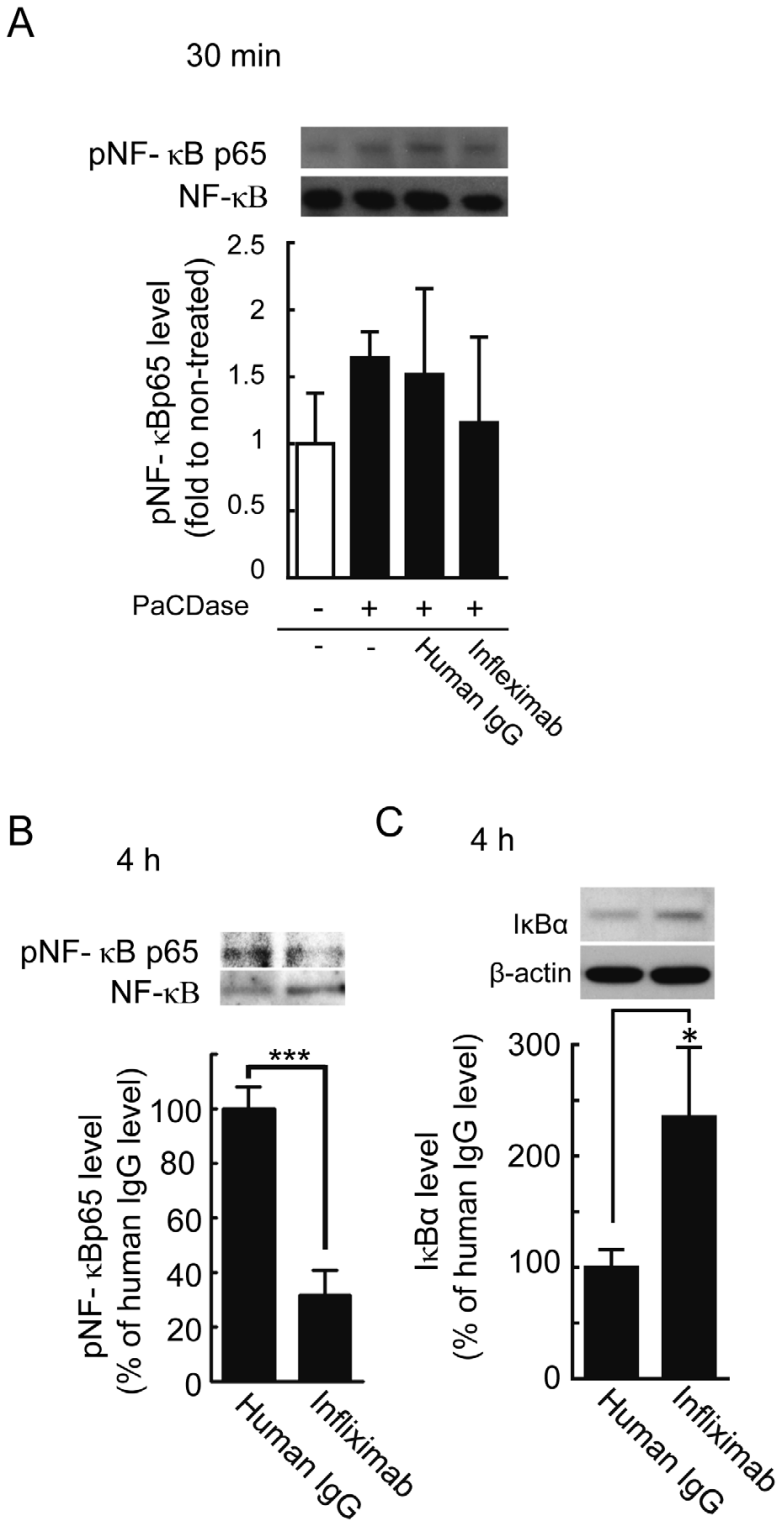
Infliximab inhibits the PaCDase-activated NF-κB-mediated signaling pathway. (*A, B*) Infliximab suppresses the phosphorylation of NF-κB p65. Nitrocellulose filters with 1 mU/ml PaCDase in the absence or presence of 100 µg/ml normal human IgG or 100 µg/ml infliximab in Tris-buffered saline with 0.1% Triton X-100 were placed on the stratum corneum. The cells were incubated for 30 min (A) or 4 h (B), washed, and solubilized. The lysates were cleared by centrifugation, and the supernatants were subjected to SDS-PAGE/ immunoblotting with anti-phospho-NF-κB p65 (Ser536) and then re-probed with anti-NF-κB p65. The blots shown represent 3 independent experiments. The band intensity of phosphorylated NF-κB p65 is shown relative to that of NF-κB p65 in each lane. The data are expressed as the ratio relative to control (-/-) and represent the mean±SD of 3 independent experiments. ***P<0.001. (*C*) Infliximab increases IκBα protein concentration in keratinocyte cytosolic extracts. Cells were incubated for 4 h as described in (A), washed and solubilized. The lysates were cleared by centrifugation, and the supernatants were subjected to SDS-PAGE/immunoblotting with anti-IκBα antibody. To determine the amount of IκBα in each band, the membranes were re-probed with anti-βactin. The blots shown are representative of 3 independent experiments. Shown is the band intensity of IκBα relative to that of βactin. The data are expressed as the ratio relative to control (-/-) and represent the mean±SD of 3 independent experiments. *P<0.05.

TNF-α has been shown to induce endothelin-1 and IL-8 production by human keratinocytes [44], [45]. TNF-α-induced IL-8 production depends on NF-κB; thus, PaCDase-induced extracellular TNF-α may stimulate IL-8 production by these cells. Epidermal keratinocytes produce and respond to TNF-α via the cognate type 1 receptor (TNFR1) [46]. Treatment with PaCDase for 4 h activated NF-κB signal transduction, while slightly, but not significantly, increasing the level of TNF-α mRNA (Fig. 6). IL-8 gene expression level was not changed by this treatment (Fig. 7). Infliximab is a chimeric IgG1κ monoclonal antibody (composed of human constant and murine variable regions) specific for human TNF-α that inhibits the TNF-α-mediated production of IL-8 in psoriasis plaques [47]. We found that incubation of 3D keratinocytes for 24 h with clinically relevant concentrations of infliximab, similar to those used in the treatment of psoriasis, suppressed the expression of IL-8, but not TNF-α, mRNA induced by PaCDase (Fig. 7). Infliximab also suppressed PaCDase-induced phosphorylation of NF-κB p65 and increased the level of IκBα protein (Fig. 8). The PaCDase-induced expression of endothelin-1 mRNA was inhibited not only by infliximab but also by normal human IgG (data not shown). We were therefore unable to determine whether infliximab inhibited the PaCDase-induced production of endothelin-1 in our 3D keratinocyte system.

## Discussion

**Figure 9:**
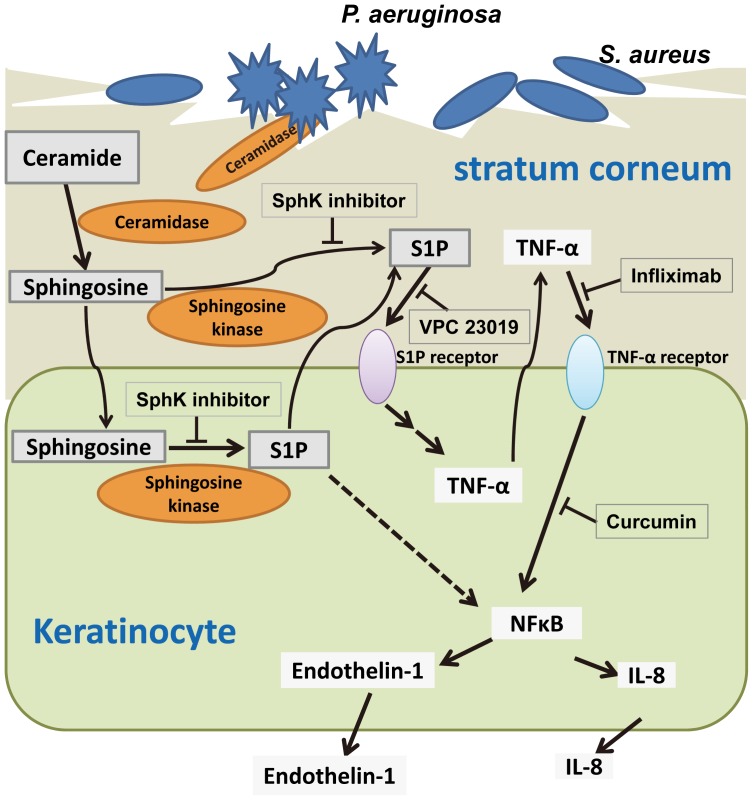
Proposed mechanism for the induction of inflammatory mediators by ceramide metabolites in human keratinocytes. PaCDase degrades ceramide into sphingosine in the damaged stratum corneum of the skin in patients with AD. Sphingosine is subsequently converted to sphingosine-1-phosphate (S1P) by SphK. S1P binds to S1P receptor 1 and/or 3, resulting in the production and release of TNF-α. The released TNF-α binds to TNF-α receptors, and IL-8 and endothelin-1 are produced via the activation of NF-κB.

We have shown here that the *Pseudomonas*-derived ceramidase, PaCDase, induced the production of inflammatory cytokines and chemokines (including TNF-α, endothelin-1, and IL-8) by keratinocytes in our 3D culture system. Among the metabolites of ceramide, S1P was responsible for the PaCDase-induced production of inflammatory mediators by the keratinocytes. A SphK inhibitor and an S1P receptor antagonist inhibited the PaCDase-induced production of the inflammatory mediators, suggesting that ceramide in the stratum corneum had been degraded by PaCDase to sphingosine and that sphingosine was in turn converted to S1P by SphK, resulting in the production of the inflammatory mediators via an S1P receptor in these cells (Fig. 9). Several anionic glycerophospholipids released from *S. aureus* by *Pseudomonas* proteases were found to enhance the PaCDase-induced hydrolysis of ceramide in the absence of detergents [12], [48]. Thus, ceramide may be degraded by PaCDase in the barrier-disrupted skin of AD patients, with the resulting ceramide metabolite S1P inducing the release of inflammatory mediators from keratinocytes.

S1P is generally considered to stimulate cells through plasma membrane G-protein-coupled receptors, *e.g.*, S1P1-S1P5 [49]. S1P was also shown to activate NF-κB independently of S1P receptors [19], [50], [51]. VPC 23019, a competitive antagonist at the S1P1 and S1P3 receptors, completely inhibited the PaCDase-enhanced gene expression of TNF-α, endothelin-1, and IL-8. Moreover, VPC 23019 inhibited the PaCDase-induced phosphorylation of NF-κB and the degradation of IκBα protein. These findings suggest that the S1P-induced production of these inflammatory mediators is regulated via S1P receptors in human primary 3D keratinocytes. Under our experimental conditions, 3D keratinocytes expressed all five types of S1P receptors. VPC 23019 acts as an antagonist at the S1P1 and S1P3 receptors, is inactive at the S1P2 receptor, and is an agonist at the S1P4 and S1P5 receptors [52]. PaCDase-induced S1P may therefore induce production of TNF-α, endothelin-1, and IL-8, primarily via S1P1 and/or S1P3 receptors in keratinocytes. In the present study, the NF-κB inhibitor curcumin inhibited PaCDase-induced expression of endothelin-1 and IL-8 mRNAs and reduced by ∼40% (not significant) PaCDase-induced expression of TNF-α mRNA. The S1P-induced production of TNF-α may by mediated in part through NF-κB-independent signal transduction pathways.

As TNF-α and TNF-dependent cytokines are involved in the immune-based inflammatory etiology of AD, TNF-α inhibitor is a plausible target for the treatment of chronic eczema [53]. Infliximab, a TNF-α-binding antibody that is used to treat patients with plaque psoriasis, psoriatic arthritis, pustular psoriasis (excluding localized type), and psoriatic erythroderma [54], has been shown to downregulate the production of anti-apoptotic proteins in regressing psoriatic skin [55]. The effects of infliximab have been evaluated in other inflammatory dermatoses and in systemic diseases involving the skin, including severe AD [56], [57]. In the present study, infliximab inhibited S1P-enhanced IL-8, but not TNF-α, production in 3D keratinocytes. TNF-α induces keratinocyte production of IL-8 via NF-κB [58]. Indeed, we found that the NF-κB inhibitor crucumin suppressed the PaCDase-induced production of IL-8. Moreover, VPC 23019 and infliximab attenuated the PaCDase-induced activation of NF-κB and the induction of IκBα protein. Thus, PaCDase-induced S1P likely induces TNF-α production and release from 3D keratinocytes via S1P receptors, resulting in TNF-α induction of IL-8 production through NF-κB-mediated signal transduction (Fig. 9).

PaCDase and S1P markedly enhanced the expression of CXC chemokines, such as IL8, CXCL1, and CXCL2, in 3D keratinocytes. Those chemokines have been reported upregulated in the lesional skin of patients with AD [38]. IL-8 content in the stratum corneum showed the highest correlation with the severity scores of AD lesions [59]. AD patients with neutrophil infiltrates showed increased concentrations of CXCL1 [60]. Thus, the PaCDase-induced production of CXC chemokines may cause acute atopic eczema through activation of leukocytes.

AD is a common pruritic, inflammatory skin disorder [61]. Chronic, localized, or generalized pruritus is a diagnostic indicator of AD. Endothelin-1 has been shown to elicit pruritis (itching) in humans [62], [63]. The molecular pathways that contribute to the transduction of itch responses to endothelin-1 do not require either neuronal PLCβ_3_ or TRPV1, which have been shown to mediate histamine- and serotonin-induced itch responses, respectively [64]. In the present study, treatment not only with PaCDase but also with S1P alone strongly induced endothelin-1 production by 3D keratinocytes, suggesting that keratinocyte-produced S1P is involved in endothelin-1-mediated pruritus in AD.

The S1P analog FTY720 (fingolimod) acts as a functional S1P receptor agonist by inhibiting the egress of lymphocytes from the thymus and secondary lymphoid organs and is therefore a promising immunosuppressant drug for the prevention of allograft rejection and for the treatment of T lymphocyte-driven inflammatory skin diseases, including lupus erythematosus, psoriasis, and AD [15]. S1P has been shown to modulate antigen capture by Langerhans cells and to inhibit the migration of these cells [16], [17]. In the present study, S1P was able to induce inflammatory responses via the production of TNF-α in human primary differentiated keratinocytes in the absence of other types of cells, *e.g.*, T lymphocytes and Langerhans cells. Keratinocytes and epidermis express all five known CDase isoforms, and the activities of acidic and alkaline CDases increase during differentiation and persist in the stratum corneum [65]. Conversely, the activities of neutral and phytoalkaline CDases predominate in proliferating keratinocytes. TNF-α is a critical cytokine in several dermatological diseases, including AD [66]. Thus, S1P may play a key role in the progression of AD, and may therefore provide a useful therapeutic target in patients with this condition.
